# Improving taxonomic resolution, biomass and abundance assessments of aquatic invertebrates by combining imaging and DNA megabarcoding

**DOI:** 10.7717/peerj.20501

**Published:** 2026-01-05

**Authors:** Philipp M. Rehsen, Mia S. Honka, Mikko Impiö, Iris Madge Pimentel, Florian Leese, Arne J. Beermann

**Affiliations:** 1 Department of Aquatic Ecosystem Research, University of Duisburg-Essen, Essen, Germany; 2 Centre for Water and Environmental Research (ZWU), University of Duisburg-Essen, Essen, Germany; 3 Department of Method Development, Finnish Environment Institute (SYKE), Helsinki, Finland

**Keywords:** Automated imaging, Machine learning, Deep learning, DNA megabarcoding, Barcoding, Biomass assessment, Biodiversity assessment, Aquatic invertebrates

## Abstract

Understanding biodiversity change requires a comprehensive assessment of not only the identity of species inhabiting an ecosystem but also their biomass and abundance. However, assessing biodiversity on the species level with precise biomass information is a time-consuming process and thus rarely applied. While DNA-based approaches like DNA barcoding offer precise species identification, they lack information on specimen size and biomass. In contrast, high-throughput imaging techniques enable rapid measurements of a specimen’s size and morphological features but may have low taxonomic resolution. In this study, we combined DNA megabarcoding, *i.e.*, high-throughput barcoding of single specimens, with semi-automated imaging and deep neural networks to produce accurate taxonomic identifications, abundance, and biomass estimations for insects. In a multiple stressor field experiment, we collected a dataset of 743 specimens from 14 species of the orders Ephemeroptera, Plecoptera, and Trichoptera (EPT), which are routinely used as aquatic biological quality indicator taxa. Each specimen was imaged, weighed, and megabarcoded using the COI barcode gene. From the images captured using the semi-automated imaging device BIODISCOVER, we curated a final dataset of 146,439 images taken from two perpendicular cameras. We trained convolutional neural networks (CNNs) with these pictures for species identification and biomass estimation and evaluated their performance. In addition, we investigated whether models pre-trained for species identification perform better on the biomass estimation task, compared to models trained solely for biomass estimation, thus potentially reducing the need for extensive labelled data in future studies. Our findings demonstrate that combining DNA megabarcoding with automated imaging and deep neural networks enables fast, reliable, but also comprehensive assessment of species composition and biomass on the specimen level, contributing to the urgently needed methods in conservation biology, ecology, and evolution.

## Introduction

Understanding biodiversity and ecosystem change necessitates the use of comprehensive biological assessment methods. For a sound understanding of why and how a system changes, and how such changes can be prevented or reverted, these assessment methods should not only include species richness and composition data, but also include information on changes in abundance and biomass (Johnson et al., 2024). Since invertebrates constitute a large part of animal diversity and biomass (Stork et al., 2015; Bar-On, Phillips & Milo, 2018; Eisenhauer, Bonn & A. Guerra, 2019), accurate estimates of invertebrate biomass are essential for quantifying community structure, food web dynamics, and energy flow in ecosystems (Wardhaugh, 2013). For invertebrates, however, biomass changes in ecosystems are typically not measured for individual species but only for complete bulk samples often containing hundreds of species and thousands of specimens (Hallmann et al., 2017; Welti et al., 2022; see also Van Klink et al., 2020). The focus on net biomass change at the community level is largely due to the lack of trained experts to identify invertebrate species along with the time and cost involved in morpho-taxonomic identification (see European Commission & Directorate General for Environment, 2022 for details). Alternative methods, such as DNA-based species identification, are able to reduce costs and scale up biodiversity assessments (Baird & Hajibabaei, 2012; Deiner, Yamanaka & Bernatchez, 2021; Chua et al., 2023; Buchner et al., 2025). Especially for samples with many specimens, these methods offer fast and precise taxonomic assignments, often down to species level. Furthermore, they are independent of the life stage or physical integrity of the specimen studied and are often the only way to distinguish cryptic species.

For DNA-based biodiversity assessments, DNA is taken either from tissue of the specimen directly or from the environment (environmental DNA). Commonly, a fragment of the DNA, a DNA barcoding region, is amplified using PCR, sequenced, and the resulting DNA sequence is compared to a reference database for species identification. DNA-based analysis of whole communities typically rely on DNA metabarcoding of homogenised bulk samples (*e.g.*, Yu et al., 2012; Buchner et al., 2025). Due to methodological limitations of metabarcording (*e.g.*, varying mitochondrial copy numbers, primer bias, polymerase chain reaction (PCR) stochasticity) this approach can neither deliver accurate abundance nor biomass information (Polz & Cavanaugh, 1998; Piñol et al., 2015; Elbrecht & Leese, 2015). As a novel alternative genetic approach, DNA megabarcoding (Chua et al., 2023) is used for identifying single specimens by employing high-throughput sequencing and is hence more cost-effective to Sanger sequencing. Although, as individual specimens are processed in contrast to complete bulk samples, it is more time consuming than DNA metabarcoding, while as a key potential allowing for assessing abundance in addition to species level identification. However, it remains unlikely that it by itself will be able to deliver exact biomass data, is it falls under the same constraints as DNA metabarcoding. Therefore, new approaches or tools are needed to comprehensively assess biodiversity change.

In comparison to DNA-based methods, image-based methods offer the possibility to assess size and morphological features used for species identification (Borowiec et al., 2022). These methods are usually based on deep neural networks (DNNs), which are trained with large amounts of data and have been shown to be powerful tools for fast species identification (Borowiec et al., 2022). Deep learning enables computational models composed of multiple processing layers to learn representations of data with multiple levels of abstraction. It can uncover complex patterns in large datasets by employing the backpropagation algorithm, which guides the adjustment of internal parameters used to transform representations from one layer to the next (LeCun, Bengio & Hinton, 2015).

Using DNNs to identify species based on images is already possible *via* publicly available mobile phone applications, particularly in the field of plants (Wäldchen & Mäder, 2018), *e.g.*, LeafSnap, Pl@ntNet or Flora Incognita (Kumar et al., 2012; Goëau et al., 2013; Mäder et al., 2021). However, reliable species identification is only possible when extensive, well-labelled training datasets are available. This is the case for the previously mentioned projects, since they focus on ‘charismatic’ groups of plants (Troudet et al., 2017).

While freshwater ecosystems are of special interest for biodiversity conservation (Dudgeon et al., 2006), only few annotated image datasets are available for freshwater invertebrates, with the biggest one being the FIN-benthic dataset (Raitoharju et al., 2018; see also Impiö et al., 2025). Additionally, among image-based studies focused on terrestrial or aquatic invertebrates, only very few included biomass data and not even for all specimens in the dataset (Ärje et al., 2020; Schneider et al., 2022). This is likely because manual species identification is a time-consuming process requiring expert taxonomic knowledge, which becomes increasingly scarce, and because of the considerable effort required to generate biomass data for individual specimens. Moreover, imaging itself can be time-consuming if done manually. While semi-automated imaging devices for plankton are already commercially available for a long time (*e.g.*, FlowCAM (Sieracki, Sieracki & Yentsch, 1998), Zooscan (Grosjean et al., 2004), or FlowCytobot (Olson & Sosik, 2007)), the development of semi-automated imaging devices for comparably larger invertebrate specimens, is an ongoing process (Ärje et al., 2020; Schneider et al., 2022; Wührl et al., 2022; De Schaetzen et al., 2023; Simović et al., 2024).

To improve DNN performance for tasks where training data are scarce, a useful technique during DNN training is fine tuning, which is based on the concept of transfer learning. Here, knowledge from different but related source domains is transferred to a specialized target domain (Pan & Yang, 2010; Weiss, Khoshgoftaar & Wang, 2016; Yin et al., 2017; Zhuang et al., 2021).

To improve biomass estimation at the level of individual specimens in conjunction with their high-resolution taxonomic identification, we investigated a new multimodal approach by combining DNA megabarcoding and image-based biomass estimation using semi-automated imaging. For this we imaged, weighed, and megabarcoded insect specimens from the orders Ephemeroptera, Plecoptera, and Trichoptera (EPT) from a recent multiple stressor outdoor experiment. EPT taxa are routinely used as biological water quality indicator taxa world-wide for biomonitoring (Resh & Rosenberg, 1989). We used both, traditional modeling approaches based on automatic image metrics (area, perimeter and max Feret diameter), and deep learning methods using solely images as input for species identification and biomass estimation. Specifically, to achieve our aim we benchmarked convolutional neural networks (CNNs) pre-trained on species labels, obtained by megabarcoding, and fine-tuned on specimen biomass prediction against (I) CNNs with identical architecture trained exclusively on biomass information, and (II) linear regression models based on pre-calculated image features. Pre-training CNNs on taxonomic specific key features like shapes, textures, colours, and structures (*e.g.*, soft-bodied *vs.* hard-bodied specimen) might provide useful information for estimating biomass.

We hypothesize that transfer learning from taxonomic information will improve CNN performance on biomass prediction, compared to both the CNN without taxonomic pre-training and the linear models. We discuss the value of the combination of novel single-specimen ‘megabarcoding’ and imaging methods for biodiversity assessments of aquatic invertebrates and beyond.

## Materials & Methods

### Sample collection *ExStream*

Benthic macroinvertebrate samples were taken from an outdoor mesocosm experiment, following the *ExStream* design of Piggott et al. (2015). The experiment was conducted from 6 March to 11 April 2022 at the Boye river in North Rhine-Westphalia, Germany (lat, lon: 51.5533 N, 6.9485 E). Field permit for conducting the *ExStream* experiment was obtained from the Lower Nature Conservation Authority, Department of the Environment and Greenery, City of Bottrop, Germany.

**Figure 1:**
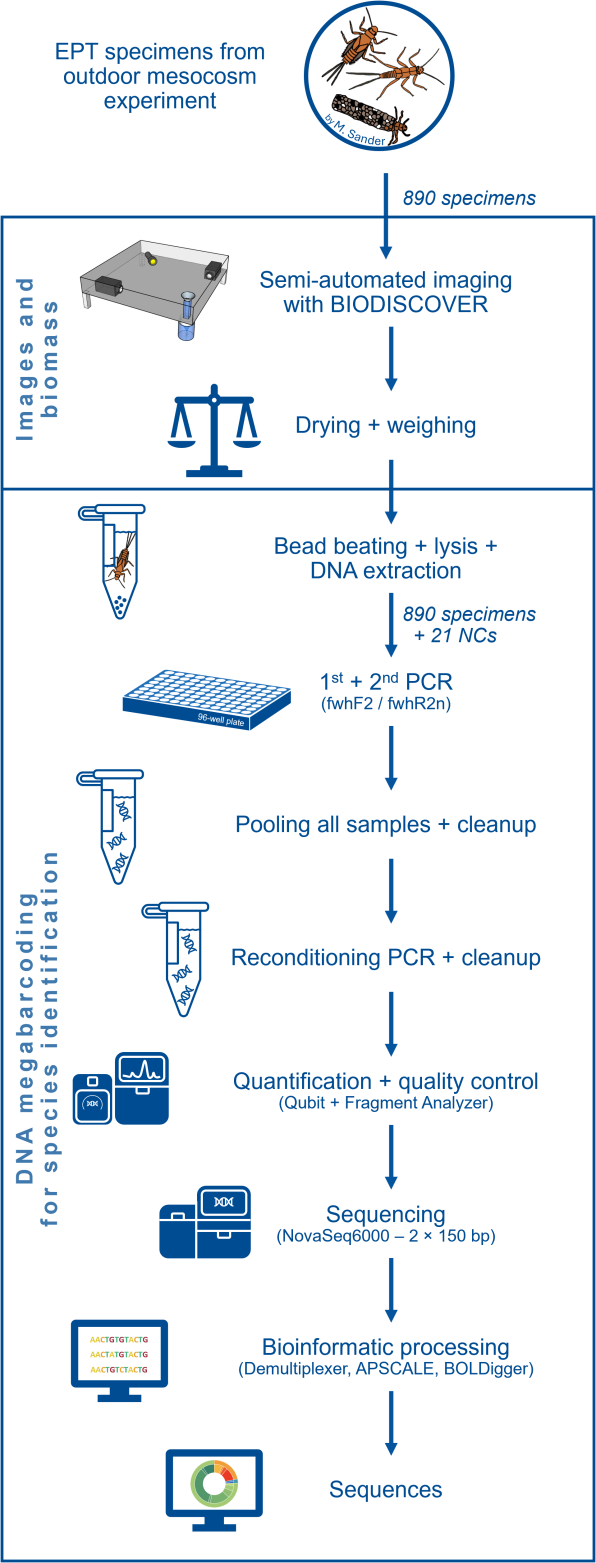
Flow diagram showing an overview of the laboratory and bioinformatic workflow. Starting with the 890 EPT specimens from an outdoor *ExStream* experiment to final DNA sequences.

Each of the 64 mesocosms of the experiment contained two habitats for MZB, namely a smaller leaf litter habitat and a larger riverbed habitat, filled with sand, gravel and stones, mimicking the riverbed of the Boye river. Riverbed habitat samples were collected in 96% ethanol and taken to the lab. After 24 h, the preservative ethanol was replaced to ensure a sufficient ethanol concentration for DNA preservation. For easier separation of specimen and sample debris, the samples were decanted (DIN EN 17136, 7.2.4). This process separates sand and gravel from organic material and simplifies the subsequent manual sorting of EPT taxa and other macroinvertebrates. During the manual sorting process, the samples were transferred into a Petri dish and examined under binoculars (EDT-101 7-45x Zoom Stereomicroscope; Bresser). EPT specimens were identified morphologically as such and stored in 96% ethanol separately. An overview over the subsequent laboratory procedures is provided in Fig. 1.

### Imaging and weighing

Images of the EPT specimens were taken with the semi-automated imaging device BIODISCOVER (Ärje et al., 2020). The device has two perpendicular cameras, directed at a cuvette filled with ethanol. When a specimen is inserted into the cuvette, it is imaged at a frame rate of 100 frames per second while sinking. Image features, such as area, perimeter, and maximum Feret diameter (*i.e.,* the longest distance between any two points along the object’s boundary), are saved for all frames. All EPT specimens were put through BIODISCOVER at least twice with the aim of collecting at least 50 images per specimen and to have sufficient training data from at least four different imaging angles. In rare cases, if a specimen sank too quickly, it was placed into the cuvette again up to a maximum of ten times. If 50 images could not be obtained after ten attempts, imaging for that specimen was concluded due to time constraints. If the casing of a Trichoptera larva rendered the specimen too large or heavy to capture even a single picture after five trials, the larva was then imaged without its casing. Additionally, where possible, Trichoptera larvae of the same species were imaged both with and without their casing to increase the diversity of the image training dataset. After imaging, the specimens were transferred into pre-weighted twist top tubes and stored in 96% ethanol until further processing. To determine the dry weight of the specimens, the ethanol was decanted, and the tubes were placed in a vacuum centrifuge (Concentrator 5301; Eppendorf) at 60 °C for at least 20 min until all ethanol was evaporated. The filled tubes were then weighed again on a precision scale (XS105 DualRange, Mettler Toledo, readability ± 0.2 mg), allowing to calculate the dry weight of the specimens by subtracting the empty weight of the tube from the total weight.

### DNA extraction

To extract the DNA of all EPT specimens individually, specimens were first transferred into 96 deep well plates for cell lysis. Deep well plates were pre-filled with six 2 mm Zirconia beads and 900 µL TNES buffer (50 mM Tris, 400 mM NaCl, 20 mM EDTA, 0.5 Mass/% volume SDS, pH 7.5) per well. After adding the specimens, 20 µL proteinase K (7BioScience, final concentration (FC) 0.217 mg/L) was added to digest proteins and nucleases, protecting the DNA from being degraded. The tissue was mechanically broken apart by five minutes of bead-beating at 2,400 rpm (Mini-Bead-Beater-96; BioSpecProducts). Lastly, the solution was incubated for 20 min at 56 °C and 1,400 rpm (ThermoMixer^®^ C, Fisherbrand). If not immediately used, the plates were stored at −20 °C until further processing.

DNA was extracted from the lysate by using a guanidine hydrochloride and ethanol-based buffer combined with silica spin column plates (Buchner, 2022a). First, GuHCl binding buffer and the lysate were combined with a 2:1 ratio (1,200 µL binding buffer and 600 µL lysate) in a 2.2 mL deep well plate. To ensure an even mixture, the plate was sealed, vortexed and shortly centrifuged. The buffer-lysate-mix was loaded onto a 96-column silica filter plate (NucleoSpin 96 Tissue Plate; Machery-Nagel) placed on a vacuum manifold. For filtering, a vacuum was applied until the entire volume had passed through the column. To wash away the non-DNA components, 600 µL of wash buffer was loaded into the wells and vacuum was applied until the wash buffer passed through the column entirely. This process was repeated once. To ensure complete removal of any residual wash buffer, vacuum was applied again for ten minutes, and the filter plates were centrifuged at 2,750 × g for one minute. To extract the cleaned DNA from the plate, 100 µL elution buffer was added to the columns. After three minutes of incubation, the plate was centrifuged at 2,750 × g and the DNA was collected in a new plate. The eluate was stored at −20 °C until the next step.

### Target gene amplification

For amplification of the target mitochondrial cytochrome c oxidase subunit I (COI) barcoding gene, we used a two-step PCR approach. During the 1st step PCR tagged versions of the fwhF2/fwhR2n primers for freshwater macroinvertebrates were used (Vamos et al., 2017). In 10 µL PCRs, 1 µL of eluate was added to a mix of 5 µL DreamTaq mastermix (DreamTaq Green PCR Master Mix, FC 1 ×; Thermo Fisher Scientific), 0.2 µL forward and reverse primer (FC 200 nM each), and 3.6 µL nuclease-free water. The used PCR protocol was chosen in accordance with the manufacturer’s recommendations and primer requirements (initial denaturation for 3 min at 95 °C, followed by 40 cycles of denaturation for 30 s at 95 °C, primer annealing for 1.5 min at 58 °C, and extension for 1 min at 72 °C, and a final extension for 10 min at 72 °C). During the 2nd step PCR, Illumina sequencing primers with additional tags were used, resulting in an individually tagged sample for each specimen (Buchner, Haase & Leese, 2021). Here, PCRs with a final volume of 10 µL were used, consisting of 5 µL DreamTaq mastermix (FC 1 ×), 1 µL forward and reverse Illumina sequencing primer (FC 100 nM each), 2 µL of the 1st step product, and 1 µL nuclease-free water (protocol: initial denaturation for 3 min at 95 °C, followed by ten cycles of 30 s denaturation at 95 °C, primer annealing for 30 s at 61 °C, and extension for 1 min at 61 °C, and a final extension for 15 min at 72 °C). PCR success was verified using gel electrophoresis. In case of no visible PCR product, PCRs were repeated using 1 µL of 1:200 diluted extracts as input. Samples of repeated PCRs were subsequently used for sequencing independent of visible PCR success after gel electrophoresis.

### Library preparation

After pooling all previously generated 1152 PCR products (890 samples + 241 repeated samples + 21 NCs), the resulting pool was cleaned up. For this, GuHCl-buffer was added in a ratio of 2:1 (seven mL GuHCl and 3.5 mL library) to the pooled library and the mixture was run through a 30 mL silica gel column with a vacuum manifold. Two times 10 mL of wash buffer were added and run through the column subsequently. To dry the column, it was centrifuged for 2,750 × g for one minute. 1,000 µL of elution buffer was added and after a 3-minute incubation period, the tube was centrifuged at 2,750 × g again. This process was repeated with a smaller silica gel column, with a starting volume of 1 mL. A total of 2 mL of GuHCl-buffer were added to the eluate and run through the silica column, followed by adding 650 µL of wash buffer twice. Finally, the DNA was eluted by adding 100 µL of elution buffer, incubated for one minute and centrifuged. The DNA concentration of the library was measured on a Qubit 4.0 following the manufacturer’s instructions.

To remove potential bubble products that have formed during PCR, a reconditioning PCR was performed (Buchner, 2024). For this, four 50 µL reactions of the reconditioning PCR were prepared with 25 µL Multiplex (FC 1 ×), 0.25 µL Illumina P5 and P7 primer (FC 0.5 µM each), nuclease-free water, and 2.2 µL template per reaction (single cycle protocol: Denaturation for 5.5 min at 95 °C, annealing for 1.5 min at 60 °C and extension for 1.5 min at 72 °C, followed by a final extension for 10 min at 68 °C). After pooling the products of the four PCRs together, an additional clean up (see above) was performed.

To remove any non-target DNA fragments, like primers and nuclear DNA, a bead-based size selection was performed (Buchner, 2022b). For this, 70 µL of clean-up beads were added to 100 µL of the cleaned-up eluate in a 1.5 mL Eppendorf tube and mixed for five minutes at 1,000 rpm on a thermomixer at room temperature to bind the DNA to the beads. After 2 min of incubation on a custom-made magnetic rack, the supernatant was discarded. Two additional washing steps were performed by covering the beads with 250 µL of wash buffer, 30 s of incubation and discarding the supernatant. After this, the beads were dried for five minutes. To elute the DNA from the magnetic beads, 50 µL elution buffer was added and mixed for five minutes at 1,000 rpm. The tube was then placed on the magnetic rack again and the supernatant was transferred to a new Eppendorf tube. The DNA concentration of the final library was measured with Qubit following the manufacturers’ instructions. Finally, we verified that all remaining DNA-fragments had the desired length of the target fragment with a Fragment Analyser (5200 Fragment Analyser System; Agilent).

### Sequencing

Samples for 890 specimens plus 21 negative controls were sequenced by GENEWIZ from Azenta Life Sciences (Leipzig, Germany) on a NovaSeq6000 (Read length: 2 ×150 bp). Raw reads are available in FASTQ format (.fastq.gz) in the European Nucleotide Archive (ENA: PRJEB94928).

### Bioinformatics

Reads were further demultiplexed with Demultiplexer (version 1.1.0—https://github.com/DominikBuchner/demultiplexer) since additional inline tags were combined with index reads. Tags were removed during demultiplexing and samples were saved with their respective imaging names (plate & well position and specimen ID) in FASTQ format. Paired-end merging, primer trimming, quality filtering, operational taxonomic unit (OTU) clustering (98% similarity threshold) and denoising, as well as LULU filtering was done with APSCALE (version 1.7.1) (Buchner, Macher & Leese, 2022). OTU sequences were queried against the BOLD database (Ratnasingham & Hebert, 2007) to assign taxonomy using BOLDigger (version 2.1.3) (Buchner & Leese, 2020). In case of several OTUs per specimen, to taxonomically assign the samples, the OTU with most reads was assigned to the specimen if consistent with the morphological identification at order level. If the PCRs for one sample with no visible bands after gel electrophoresis were repeated using diluted templates, the specimen was taxonomically assigned based on the sample with more reads. Assignments with less than 1,000 reads were discarded. The resulting top hit OTU table was merged with the metadata gathered by the BIODISCOVER imaging device and the specimen weights.

### Data pre-processing

To ensure high quality of the dataset, all images taken by the BIODISCOVER imaging device were manually inspected and images with non-target objects such as bubbles, forceps, and debris were removed.

The dataset was then split into train, test and validation sets in five folds by using the stratified K-Fold iterator with non-overlapping groups from Scikit-learn (Pedregosa et al., 2011). This approach ensures an equal distribution of specimens from the same species across the folds.

For some of the individuals, a taxonomic expert performed a plausibility check of the DNA-based taxonomic assignment. This was done for (1) the 100 individuals, which most frequently showed a disagreement between CNN predictions and DNA-based identification for all five folds during initial CNN training in several pre-tests, (2) for the six least abundant species, and (3) for 100 randomly selected individuals. Taxonomic assignments were corrected in three cases, and four samples for which taxonomic identification could not be ensured were excluded from the dataset.

For the final dataset, EPT species with fewer than five specimens were excluded to ensure that images from at least one specimen were represented in each of the five cross-validation folds. After data cleaning, the train, test, and validation splits were re-calculated. All images were then resized to 224 × 224 pixels while keeping the aspect ratio across all images globally to maintain the size ratios of the individuals important for biomass (Fig. S1). The cross-validation folds are the same for both species identification and biomass estimation tasks to make the results as comparable as possible.

### Species identification models

Taxonomic classifiers were trained on different taxonomic levels (species (*n* = 14), genus (*n* = 13), family (*n* = 7), and order (*n* = 3)), to train the CNNs on taxonomically relevant features. This idea is derived from the concept of transfer learning, where a pre-trained model can leverage knowledge gained from out-of-domain data to improve performance on another task (*e.g.*, pre-training CNNs on ImageNet data).

For each taxonomic level, CNNs with ResNet18 (He et al., 2016) and EfficientNet-B0 (Tan & Le, 2019) architecture were trained on an NVIDIA Tesla V100-GPU using the AdamW optimizer (Loshchilov & Hutter, 2019), cross-entropy loss function and TrivialAugment (Müller & Hutter, 2021) for augmentation. The best learning rate over all five folds was determined using lr_find from PyTorch Lightning (Falcon & PyTorch Lightning team, 2025) and subsequently used to train the networks for 100 epochs. To obtain the model with the least overfitting, the validation loss was evaluated after each epoch and only the best performing model was saved.

### Biomass estimation models

The best performing CNNs pre-trained on taxonomic information were then used as a starting point to train CNNs for biomass prediction. Since the efficientnet_b0 model outperformed the ResNet18 model for classification on all taxonomic levels, only the EfficientNet models were used for fine tuning on biomass. After loading the weights of the best performing model, CNNs were trained with L1 loss and flips and rotations as augmentations, while keeping the aspect ratio of the image. The best learning rate over all five folds was determined using lr_find from PyTorch Lightning and subsequently used to train the networks for 10 epochs. This was done with frozen and not frozen bases (*i.e.,* either keeping the base layer weights fixed and only training the final linear projection layer or allowing the base layers to be updated during training) targeting weights, log transformed, and log +1 transformed weights and all combinations of these. For biomass prediction without taxonomic assignment, CNNs with efficientnet_b0 architecture were trained the same way, but for 100 epochs starting with imagenet weights.

### Model evaluation

As a baseline to evaluate CNN performance, ordinary least squares (OLS) linear regressions based on the measurements collected by the BIODISCOVER imaging device were calculated using Scikit-learn. Specifically, we fit linear models with predictors area (A), maximum feret diameter (MFD) and perimeter (P). We denote each model with linear (x,y,z), where x, y and z represent the predictors used to fit the model. For example, linear (A,MFD) represents a model fitted area and maximum feret diameter.

After CNN training, each cross-validation fold’s best-performing model was used to predict on its corresponding hold out data using non-augmented images separately. This allowed the usage of each sample for predicting species identity or biomass at least once and subsequently calculating performance metrics for the complete dataset for the used predictor settings (*i.e.,* different taxonomic pre-trainings, different weight transformations, frozen/unfrozen base *etc.*). The sets were then combined to produce a final prediction set corresponding to the full dataset. This jackknife-style combining of the cross-validation folds reduces the variance of the performance metric estimates with smaller datasets, as metrics are calculated for the complete dataset.

For evaluating classification CNNs, standard CNN performance metrics were used for model comparison, namely precision, recall and F1-score. Precision is the number of true positives divided by the total number of positive predictions (true and false negatives). Recall is the number of true positives divided by the total number of actual positive classes (true positives and false negatives). F1-score is the harmonic mean of precision and recall. Additionally, for evaluation of the biomass predicting CNNs, coefficient of determination (R2), mean absolute error (MAE), median absolute error (MdAE), as well as mean absolute percentage error (MAPE), and median absolute percentage error (MdAPE) were calculated.

## Results

### Weighing

**Figure 2:**
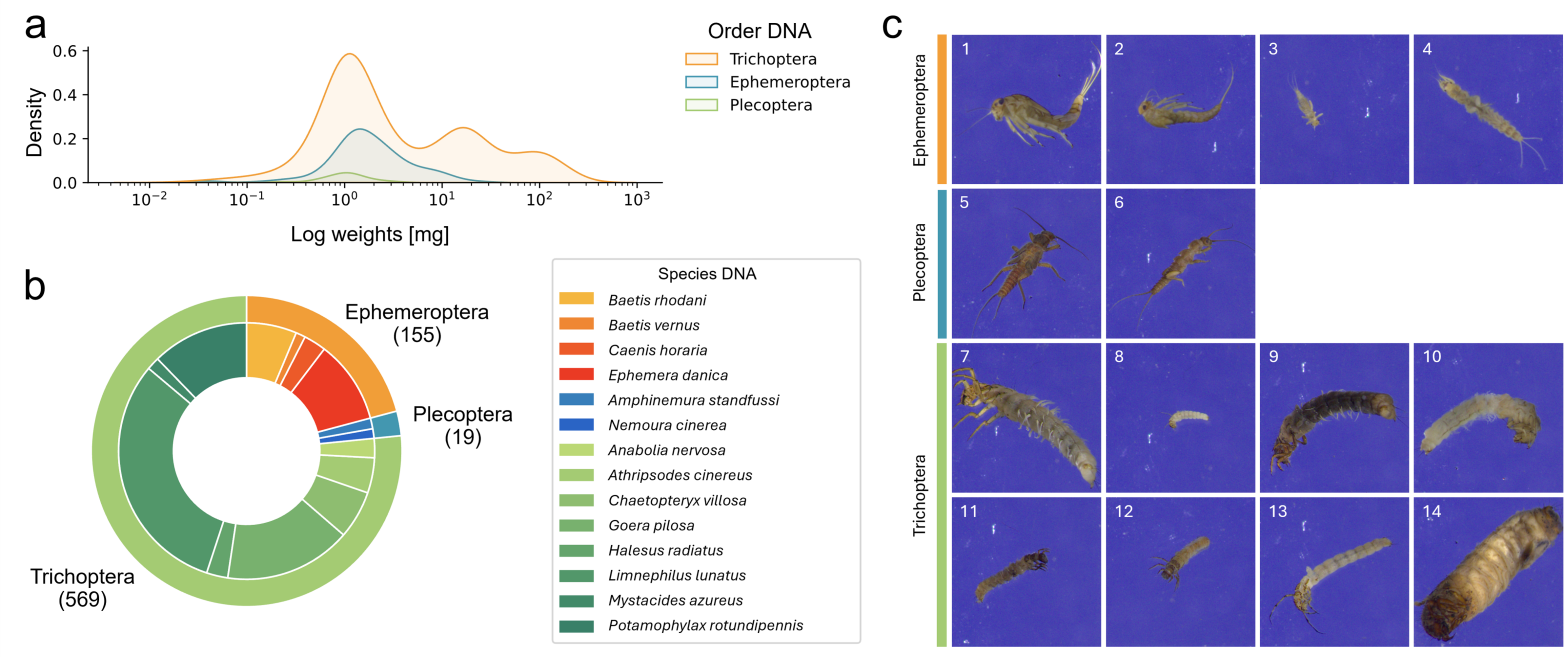
Overview over the final EPT-14 dataset. (A) KDE-plot showing the weight distribution of specimens. (B) Proportion of EPT specimens on species (outer circle) and order (inner circle) level (*n* = 743). (C) Example images from the EPT-14 image dataset. Ephemeroptera species: 1: *Baetis rhodani*, 2: *Baetis vernus*, 3: *Caenis horaria*, 4: *Ephemera danica*, Plecoptera species: 5: *Amphinemura standfussi*, 6: *Nemoura cinerea*, Trichoptera species: 7: *Anabolia nervosa*, 8: *Athripsodes cinereus*, 9: *Chaetopteryx villosa*, 10: *Goera pilosa*, 11: *Halesus radiatus*, 12: *Limnephilus lunatus*, 13: *Mystacides azureus*, 14: *Potamophylax rotundipennis*. The images shown are directly coming from the BIODISCOVER imaging device. Original images as well as modified input images for the CNNs (image size reduced to 224 ×224 pixels, keeping the aspect ratio across all images by filling the borders) can be found in Zenodo.

We determined the weight of 890 specimens, which ranged from 0.02 to 211.63 mg after excluding five small specimens with weights below the detection limit. A large proportion of specimens (23.8%) weighed less than 1 mg (see Fig. 2A for weight distribution in the final dataset).

### DNA

Sequencing resulted in 266,084,013 raw reads. Reads from 809 of the 890 samples passed the quality control, resulting in a total of 211,969,611 reads after bioinformatic processing, which clustered into 5,501 OTUs. After taxonomic filtering for macroinvertebrates, each sample had an average of 205,284.15 reads (± 124,729.49 reads, median 232,114.50 reads), assigned to on average 12.72 (± 19.46) OTUs. Samples on plate 6 showed a higher number of OTUs compared to other processed plates (4,924 OTUs compared to an average of 353 (± 125) OTUs per plate). Reprocessing and sequencing these samples confirmed that contamination was mainly attributed to non-EPT taxa. In cases where a high number of reads was associated with more than one EPT taxon, pictures were used to cross-validate the identification, ultimately confirming the taxonomic annotation. For each specimen, the assigned dominant OTU had on average 225,328.86 reads (± 92,923.84 reads, median 235,049.50 reads). 768 (*i.e.,* 86.3%) of the studied specimens were assigned to a total of 20 EPT species. In 64 (7.2%) cases, sequencing did not return enough reads, in 30 (3.4%) cases, samples were removed since they did not match the morphological identification to order level, and in 28 (3.1%) cases, hits were not returned on species level by the database.

### Imaging

We generated 184,851 images with the BIODISCOVER device of which 170,436 passed the quality control. For the final dataset, six EPT species with fewer than five specimens were excluded. This ensured that images from at least one specimen were represented in each of the five cross-validation folds. Thus, the final dataset consists of 743 specimens from 14 EPT species, hereafter referred to as the EPT-14 dataset (Fig. 2), with a total of 146,439 images.

### Pre-training on species identification

**Figure 3:**
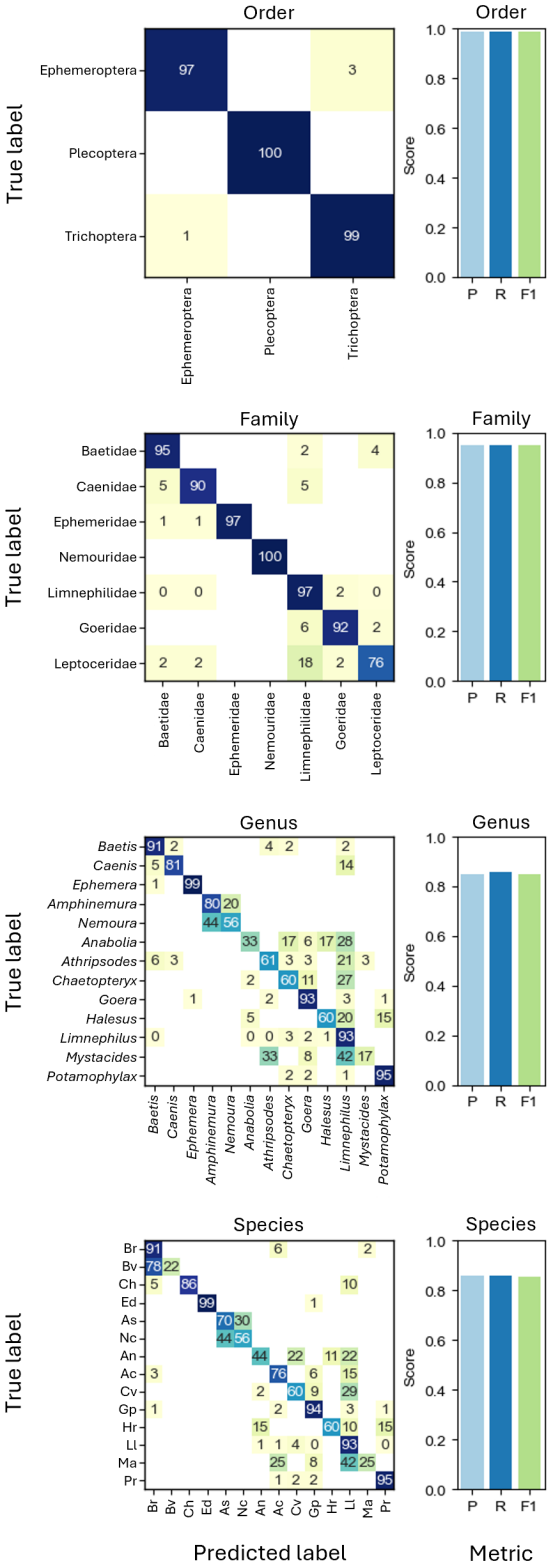
Confusion matrices showing the grouped prediction results of the CNNs trained on different taxonomic levels. Confusion matrix numbers represent percentages of true values with rows sum to 100. Cells with zeros represent nonzero values rounding to zero. Bar charts show CNN performance metrics weighted precision (P), weighted recall (R), and weighted F1-score (F1) for each taxonomic level. Abbreviations of species names as shown in the axis labels of the species CNN confusion matrix: *Baetis rhodani* (Br), *Baetis vernus* (Bv), *Caenis horaria* (Ch), *Ephemera danica* (Ed), *Amphinemura standfussi* (As), *Nemoura cinerea* (Nc), *Anabolia nervosa* (An), *Athripsodes cinereus* (Ac), *Chaetopteryx villosa* (Cv), *Goera pilosa* (Gp), *Halesus radiatus* (Hr), *Limnephilus lunatus* (Ll), *Mystacides azureus* (Ma), *Potamophylax rotundipennis* (Pr).

Based on taxonomic assignments from DNA megabarcoding, CNNs were trained on different taxonomic levels, *i.e.,* order (three groups), family (seven groups), genus (13 groups) and species level (14 groups) with all cross-validation folds. In all tests, performance metrics of CNNs using EfficientNet-B0 were consistently higher than those using ResNet18 for the EPT-14 dataset and will only be shown from here on (Fig. S2). The best performing taxonomic classification CNNs were the ones trained on order level, which could correctly predict the order of previously unseen individuals in 98.92% of cases (weighted F1-score = 0.9892, Fig. 3).

### Biomass prediction

For biomass prediction, we first applied linear models based on automatic image measurements taken with the BIODISCOVER device. The linear model based on perimeter performed worse than all other tested predictors with a median absolute percentage error (MdAPE) of 4.54 and a median absolute error (MdAE) of 8.03 mg. The best performing linear model was the one based on all predictors, namely log transformed measurements of area, maximum Feret diameter and perimeter simultaneously (MdAPE_linear (A,MFD,P)_ = 0.39, MdAE_linear (A,MFD,P)_ = 0.72). Detailed results and further metrics can be found in Table S3.

**Figure 4:**
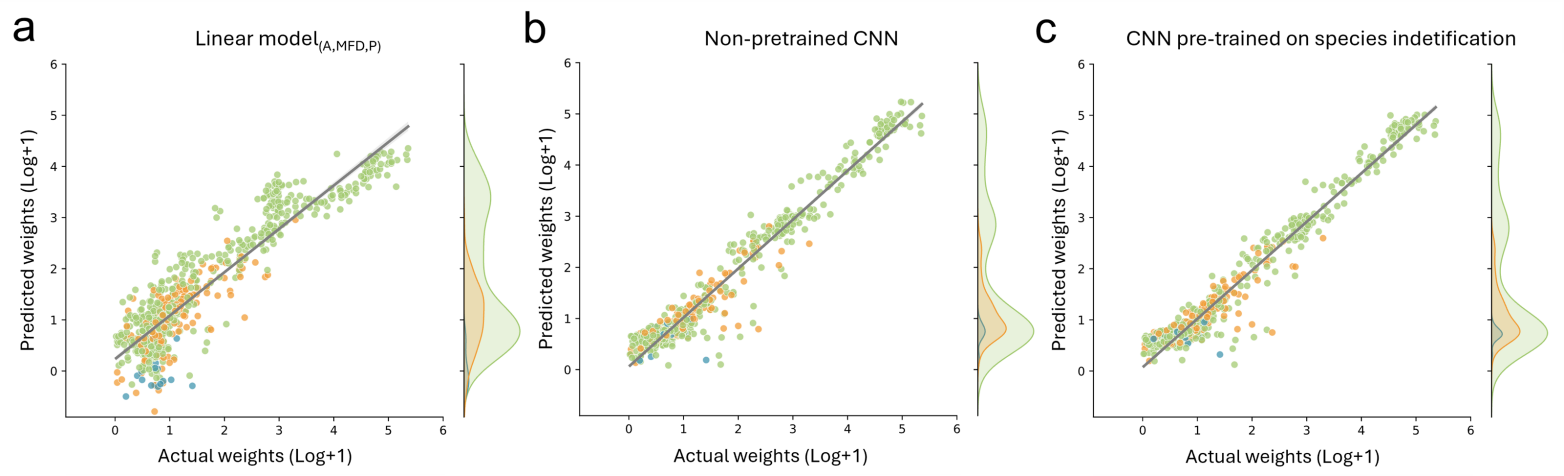
Comparison of biomass predictions. Results from the best performing (A) linear model based on automatic image measurements (area, max Feret diameter, perimeter) taken by the BIODISCOVER device, (B) non pre-trained CNN, and (C) CNN pre-trained on species identification. For this figure the predictions for all five CNNs (one per fold) are summarised, allowing the usage of each sample for predicting biomass at least once.

Additionally, we fine-tuned CNNs, pre-trained on taxonomic information with frozen and unfrozen bases, on biomass prediction and we trained CNNs without taxonomic pre-training to predict biomass. CNNs which were pre-trained on different taxonomic levels and fine-tuned on biomass with frozen bases performed better than the best performing linear model for all metrics except MAE, where only the CNNs pre-trained on genus level information achieved better results (MAE_CNN (genus)_ = 5.77 compared to MAE_linear (A,MFD,P)_ = 6.73). However, CNN performance with unfrozen bases was better in all cases for all tested metrics. Performance was best when using log+1 transformed data. Finally, the CNNs without pre-training relying on log+1 transformed weights performed similarly well for MAE and yielded best results for all other tested metrics (Table 1, Fig. 4 and Table S3).

## Discussion

We investigated a new multimodal approach by combining DNA megabarcoding and image-based methods for assessing species diversity, abundance, and biomass of benthic macroinvertebrates. Presently, the collected dataset is to the best of our knowledge the largest publicly available fully multimodal dataset with images, DNA barcode, and biomass for all specimens. This dataset contributes to closing the gap of missing comprehensive freshwater invertebrate datasets as a major bottleneck for ecological research. While species identity and abundance data were successfully derived from DNA megabarcoding, biomass was predicted reliably using a semi-automated imaging approach.

### Species identification with DNA megabarcoding

The taxonomic assignment and corresponding abundance of the specimens *via* DNA megabarcoding was successful and an identification on species level was possible for 85.6% of the samples. Since species assignment of sequences is highly dependent on the completeness of reference databases, particularly with respect to the sampled organism group and their geographic origin (Keck, Couton & Altermatt, 2023), it is more appropriate to evaluate and compare performances based on the sequence recovery rate, *i.e.,* the percentage of specimens yielding a taxonomically valid read after bioinformatic processing. Previous studies have reported sequence recovery rates of 85.21% (*n* = 1,136, Srivathsan et al., 2018), 81.15% (*n* = 8,699, Srivathsan et al., 2019), 78.45% (*n* = 3,973, pooling four to ten specimens, De Kerdrel et al., 2020), and 92.14% (*n* = 100,320, Hebert et al., 2025). The two latter studies also reported significant variation in recovery success across taxonomic orders, but only the Barcode 100 K (Hebert et al., 2025) dataset included EPT taxa, with reported success rates of 78% for Trichoptera (*n* = 444) and 8% for Ephemeroptera (*n* = 48). In comparison, we achieved a sequence recovery rate of 90.9% (*n* = 890), ranging among the highest reported values. Since the majority of the failed samples did not return any reads from sequencing despite having sufficient organic input material, we hypothesize that PCR amplification was inhibited and suggest using inhibitor removal protocols in future studies (Buchner, 2023; Schrader et al., 2012).

**Table 1:** Performance metrics of predictors trained with log+1 transformed weight. For all pre-trained CNNs the results for fine tuning with unfrozen weights are shown, since they performed better than the CNNs fine-tuned with frozen weights. Best results are highlighted in bold. An overview with all results can be found in Fig. S3.

Predictor	MdAPE	MAPE	MdAE	MAE	R^2^
**Linear model**: Area + MaxFeretDiameter + Perimeter	0.487	0.967	0.964	7.270	0.606
**CNN**: Pre-trained species	0.175	0.508	0.393	**2.492**	0.929
**CNN**: Pre-trained genus	0.173	0.506	0.377	2.624	0.925
**CNN**: Pre-trained family	0.179	0.487	0.406	2.866	0.905
**CNN**: Pre-trained order	0.191	0.488	0.407	3.501	0.870
**CNN**: Without pre-training	**0.171**	**0.444**	**0.366**	2.621	**0.930**

### Image-based CNNs—species classification and biomass estimation

In our study, image-based CNNs trained on taxonomic classification correctly assigned 86.0% of specimens to species level and 98.9% on order level. Previous studies using image-based CNNs for taxonomic identification show that classification accuracy depends on the chosen grouping. For example, Høye et al. (2022) report an identification success of up to 99.2%, however, using mixed taxonomic levels of species and genus. Our results show that, especially for closely related taxa such as *Baetis rhodani* and *Baetis vernus*, species level identification based on 224  ×  224 pixel images is challenging (see Fig. 3). Notably, when resizing images to a fixed target size while preserving the original aspect ratio to include large specimens without distortion can complicate precise species classification. Specifically, this process further reduces the size of already small specimens, causing them to occupy a smaller proportion of the image and increasing the amount of background, potentially causing relevant morphological features to be lost or less visible (Fig. S1).

However, since we focused on biomass prediction, we also show that pre-training with classification CNNs on higher taxonomic levels (*e.g.*, trained on order and family level) does not improve biomass prediction performance metrics, even though they were highly accurate in taxonomic classification. This is in contrast to our hypothesis and unexpectedly the CNNs without taxonomic pre-training achieved a predictive performance of R2 of 0.93, which is similar to the CNNs pre-trained on species level with unfrozen weights (R2 = 0.929). Notably, we found that the performance of pre-trained CNNs without frozen weights increases when increasing the label granularity during pre-training (Table S3).

The bad performance with frozen weights is potentially explained by a mismatch between the CNNs taxonomically relevant morphologic features and those important for biomass estimation, thereby impairing their generalisation performance. The features learned during the species identification task are not general enough for a projection to biomass value. Nonetheless, the good performances of the CNNs trained exclusively on biomass show that the system can learn complex relationships without the need of labour-intensive species labelling, allowing to speed up the training of biomass predicting CNNs.

The weights of some specimens fell below the detection limit of our precision scale, which became evident through records of negative weights and led to their exclusion from the dataset. Such systematic errors during weighing can cause downstream issues, since incorrect ground truth weights can have a large effect on the results of biodiversity studies. Since weighing light specimens requires an even more precise scale and is thus a more time-consuming process, it strengthens the benefit of biomass estimation based on image-based methods. Once a dataset has been compiled through the labour-intensive process of weighing small specimens, a CNN can be trained on these data. The resulting model can then be applied for further samples and used for faster but still precise biomass prediction.

### Future steps of megabarcoding and imaging

Future studies should aim to improve the success rate of species identification *via* DNA megabarcoding. With additional laboratory working steps, such as the suggested inhibitor removal protocol, one might consider using automated liquid handlers which at the same time would enable upscaling and standardisation, since laboratory protocols in our megabarcoding workflow are already suited for automation (Buchner et al., 2021; Wührl et al., 2022; Meier, Lawniczak & Srivathsan, 2025). This potential of automation marks a clear advantage for species identification *via* DNA megabarcoding over manual identification. However, the method is only as good as its reference database and will only improve with increasingly comprehensive databases (Keck, Couton & Altermatt, 2023).

The imaging of single specimens is fast with the BIODISCOVER machine, however, it requires the researcher to manually insert every specimen into the cuvette individually. The automation of this process to image single specimens from bulk samples without manual intervention has already been addressed in some projects (Wührl et al., 2022; Wührl et al., 2024). Data curation was still done manually in these projects to ensure that images used for training represent only full-body representations of specimens without foreign objects. Further automation of dataset curation can also make this sorting process significantly more efficient (Impiö, Rehsen & Raitoharju, 2024) and will be easier the more datasets are available.

It should be noted that classifiers usually assume a closed-set setting, where predictions are made on classes available during training, meaning they cannot identify previously untrained species. Recently, increasing attention has been directed towards open-set scenarios, which more accurately reflect real-world conditions in biodiversity assessment, as they allow for the introduction of previously unseen classes at a later stage (Chen et al., 2025; Yang et al., 2024). However, since DNNs proved effective for biomass assessment even without taxonomic information, we see high potential for individual-level biomass estimation independent of taxonomic assignments. While this seems trivial in the first place, size-structure in biological communities may also be an important indicator of ecological change. The (un)evenness of biomass among individuals can for example serve as a proxy for food-web complexity. This enables the application of taxonomy-free DNA-based approaches, which do not rely on assignment *via* reference databases, but analyse sequences directly without any taxonomic association (Apothéloz-Perret-Gentil et al., 2017; Mächler, Walser & Altermatt, 2021). Future studies should include further invertebrate species and test the limits of generalisation of biomass predicting CNNs.

### Added value of the combination/transferability

The combination of both methods opens vast possibilities to tackle spatial and topical imbalances in biodiversity research (Tydecks et al., 2018). This is because it offers fast and cheap integrative biodiversity assessments: On individual level, a DNA-based taxonomic assignment, reference images for the specimen, and data on biomass can be obtained. In our dataset, several specimens yielded additional OTUs, whose taxonomic assignment did not fit the morphological assignment to the EPT orders. We suspect that these OTUs represent parasites and gut content. Therefore, we highlight the potential of applying DNA megabarcoding with a high sequencing depth and the use of additional primers to investigate ecological interactions between parasites and hosts, predator and prey, or consumer and resource. This would allow a more holistic way of studying ecosystems and food webs.

Also, damaged specimens can be assigned *via* DNA megabarcoding, since usually extracting DNA from one leg is sufficient to receive a useful DNA sequence by barcoding. Images from these damaged specimens might also be used for training identifiers, potentially allowing to gain more information than with morphological identification.

With additional tests and/or training, this new combined approach to evaluate ecosystems could also be applicable for different taxa of different realms (terrestrial, limnic, marine). With the setup used, the only limitation is the size of the cuvette for imaging (1  × 1 cm) and the size of the wells of the plates used for DNA extraction of specimens (0.84  ×  0.84 cm). Labware could easily be exchanged and adjusted depending on the size of the specimens, and the BIODISCOVER machine could be modified for bigger specimens by changing the cuvette and adjusting the cameras accordingly. For imaging of smaller specimens, however, more adjustments of BIODISCOVER would be required, since different cameras or lenses would be needed. To image specimens below 1 mm in body size, one might consider using already existing commercially available imaging devices such as the FlowCAM or ZooScan. The current setup is, however, already broadly applicable, allowing to cover a wide range of taxa (Ärje et al., 2020). Currently, for samples yielding high numbers of specimens such as Malaise traps, the approach is limited by the manual steps within the laboratory workflow. While proof-of-principle studies already tested further automatization of specimen handling (Ascenzi et al., 2025; Wührl et al., 2024), they still need to be validated in large-scale projects.

If such devices and workflows are engineered to be affordable, it facilitates the integrated application of DNA-based and image-based methods and enables comprehensive assessment of understudied areas and habitats across different taxa, especially in biodiverse global south countries.

## Conclusions

In conclusion, by combining automated imaging with DNNs, reliable biomass data can be obtained even without taxonomic pre-training. Since species-level image classification was found to be challenging especially for closely related taxa, we suggest coupling the image-based biomass assessment with megabarcoding, since this approach enables acquiring individual-level biomass information with high taxonomic resolution, potentially including inferences on parasites and gut content composition.

Thus, to overcome the bottleneck of manual data labelling for supervised learning of image-based methods, we suggest combining them with DNA-based species identification. Combining both methods could help finding new bioindicator species and enhance the evaluation of ecosystem health, including both species abundance and biomass. This enables us to investigate why and how our ecosystems change.

##  Supplemental Information

10.7717/peerj.20501/supp-1 Supplemental Information 1 Comparison of original images as captured by the BIODISCOVER machine (left) and resized images as used for CNN input (right) Images with and without keeping the aspect ratio across all images globally are shown, i.e., resizing all images in the dataset while preserving each image’s original width-to-height pixel ratio in respect to the largest image of the dataset. Padding with randomly sampled pixels from the image border was applied to reach the required input dimesons. Original images, as well as padded and resized images are available on Zenodo.

10.7717/peerj.20501/supp-2 Supplemental Information 2 Overview over performance metrics from classification CNNs. using the EfficientNet-B0 (left) and ResNet18 (right) architecture targeting different taxonomic levels of the EPT-14 dataset Note: *Y*-axis labels may differ across subfigures.

10.7717/peerj.20501/supp-3 Supplemental Information 3 Extended overview of performance metrics of predictors Included are trainings with frozen and not frozen bases, targeting weights, log transformed, and log +1 transformed weights and all combinations of these for the EPT-14 dataset. Best results are highlighted in bold.

## Additional Information and Declarations

### Field Study Permissions

The following information was supplied relating to field study approvals (*i.e.*, approving body and any reference numbers): Field experiments were approved by the Lower Nature Conservation Authority Department of the Environment and Greenery Division of the City of Bottrop.

### DNA Deposition

The following information was supplied regarding the deposition of DNA sequences: The raw sequence data are available at ENA: PRJEB94928

### Data Availability

The following information was supplied regarding data availability: Data is available at Zenodo: Rehsen, P. M., Honka, M. S., Impiö, M., Madge Pimentel, I., Leese, F., & Beermann, A. (2025). EPT-14 Dataset: A Multimodal Dataset for Fine-Grained Species Classification and Biomass Prediction of Aquatic Macroinvertebrates. University of Duisburg-Essen. https://doi.org/10.5281/zenodo.15966820
The raw sequence data are available at ENA: PRJEB94928
Code is available at GitHub and Zenodo: - https://github.com/PhilippMRehsen/EPT-14_code
- Philipp M. Rehsen. (2025). PhilippMRehsen/EPT-14_code: EPT-14_code_submit (v1.0). Zenodo. https://doi.org/10.5281/zenodo.17397294
