# Supplementary figures and images for: Improving taxonomic resolution, biomass and abundance assessments of aquatic invertebrates by combining imaging and DNA megabarcoding
Source: PeerJ. 2026 Jan 5;14:e20501. doi: 10.7717/peerj.20501 (PMC12782037; doi:10.7717/peerj.20501)

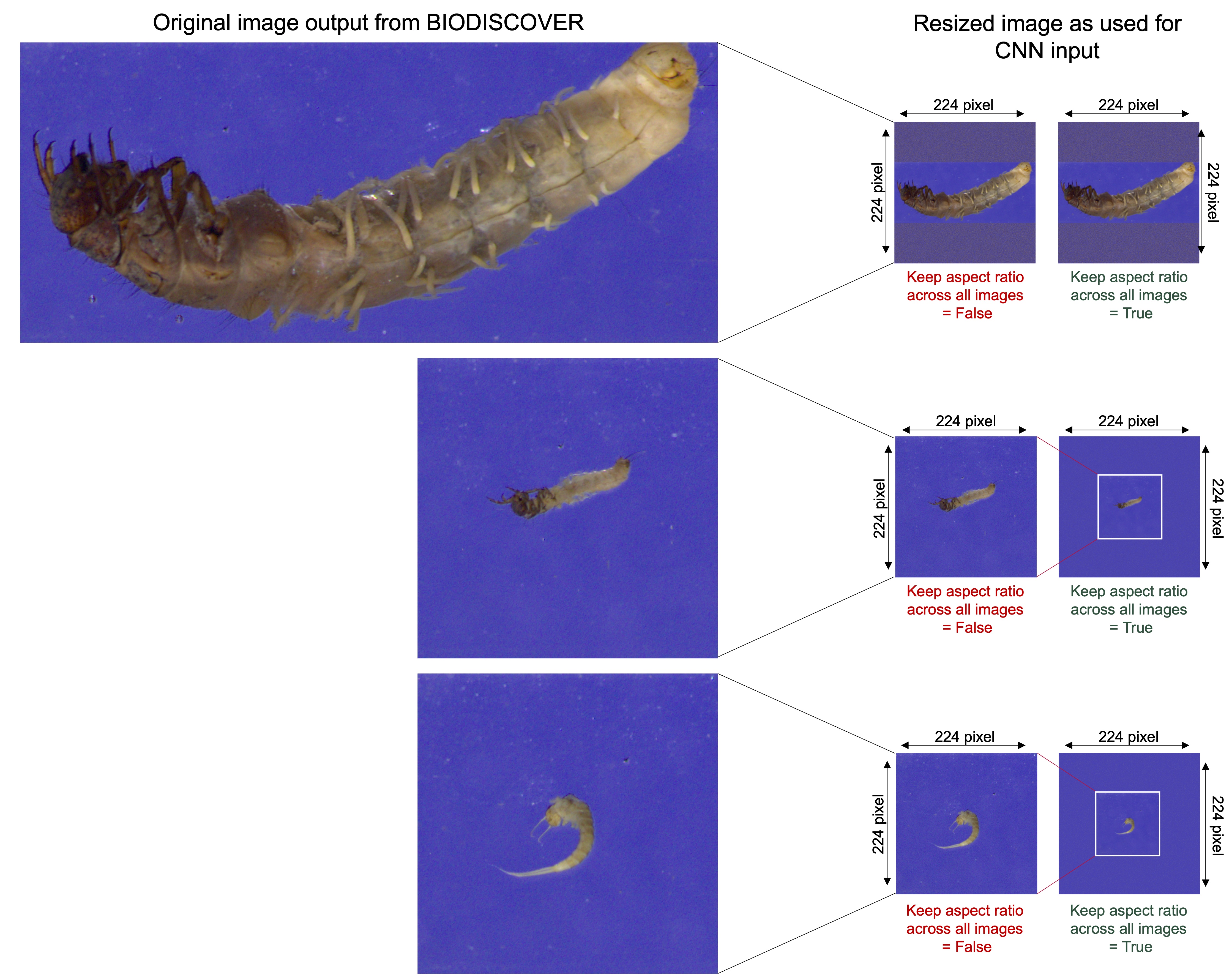

Supplement: Supplemental Information 1 — Images with and without keeping the aspect ratio across all images globally are shown, i.e., resizing all images in the dataset while preserving each image’s original width-to-height pixel ratio in respect to the largest image of the dataset. Padding with randomly sampled pixels from the image border was applied to reach the required input dimesons. Original images, as well as padded and resized images are available on Zenodo. [file peerj-14-20501-s001.png]

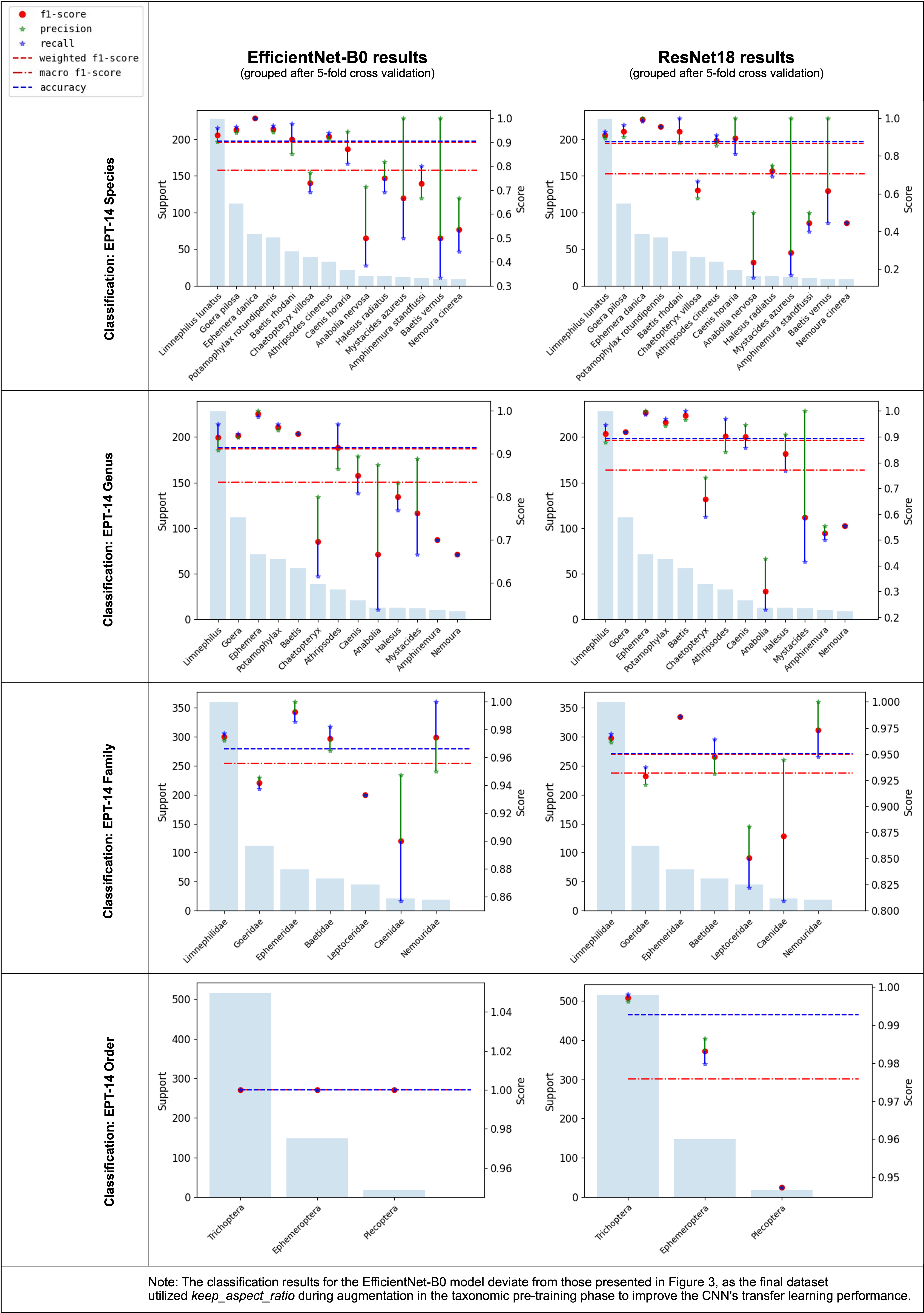

Supplement: Supplemental Information 2 — Note: Y-axis labels may differ across subfigures. [file peerj-14-20501-s002.png]
