# Supplementary material for: Improving taxonomic resolution, biomass and abundance assessments of aquatic invertebrates by combining imaging and DNA megabarcoding
Source: PeerJ. 2026 Jan 5;14:e20501. doi: 10.7717/peerj.20501 (PMC12782037; doi:10.7717/peerj.20501)
Supplement: Supplemental Information 3 — Included are trainings with frozen and not frozen bases, targeting weights, log transformed, and log +1 transformed weights and all combinations of these for the EPT-14 dataset. Best results are highlighted in bold. [file peerj-14-20501-s003.docx]

**Table S3: Extended overview of performance metrics of predictors.** Including training with frozen and not frozen bases, targeting weights, log transformed, and log +1 transformed weights and all combinations of these for the EPT-14 dataset. Best results are highlighted in bold.
